# Common Hematologic Emergencies—Acute Promyelocytic Leukemia and Microangiopathic Hemolytic Anemias—A Pivotal Role of Clinical Laboratory

**DOI:** 10.1111/ijlh.14531

**Published:** 2025-07-16

**Authors:** Ganna Shestakova, Nicolas Ulrich Edgar, Anton V. Rets

**Affiliations:** ^1^ University of Utah Department of Pathology Salt Lake City Utah USA; ^2^ ARUP Laboratories Salt Lake City Utah USA; ^3^ University of Utah School of Medicine Salt Lake City Utah USA

**Keywords:** acute promyelocytic leukemia, APL, hematologic emergency, schistocytes, thrombotic microangiopathic anemia

## Abstract

Hematologic emergencies are urgent health conditions which result in significant mortality and morbidity unless timely therapeutic measures are taken. Therapeutic success depends on their timely and accurate recognition by hematology laboratory services. This review highlights the laboratory's role in identifying conditions associated with increased schistocytes (thrombotic microangiopathies) and acute promyelocytic leukemia. Their detection based on the routine laboratory and morphology methods should trigger appropriate additional laboratory workup including molecular tests, flow cytometry, etc. Hematology laboratory professionals should be skilled to recognize these emergencies and recommend additional workup, playing a critical role in the timely diagnosis and management of life‐threatening conditions.

## 1 Introduction

A complete blood count (CBC) is one of the top five, if not the most ordered laboratory tests [1]. A CBC's importance as a diagnostic staple puts hematology laboratory services in a unique position of being the first to recognize many pathologic conditions. Given this, laboratory scientists and pathologists commonly encounter situations where rapid and correct diagnoses are critically important. Emergencies in hematology and oncology practice present with regularity from a multitude of settings, ranging from general providers' offices and urgent care to highly specialized oncology hospitals. These hematologic emergencies are urgent health conditions which result in significant mortality and morbidity unless timely therapeutic measures are taken. Therefore, these emergencies constitute actionable situations, and successful management depends on their timely, accurate recognition by hematology laboratory services.

From a laboratory medicine perspective, hematologic emergencies may include scenarios associated with markedly abnormal test results, such as metabolic emergencies (hypercalcemia, tumor lysis syndrome, hyperkalemia, hyperviscosity associated with monoclonal proteinemia). In the hematology laboratory, encountered conditions include hyperleukocytosis/leukostasis, microorganisms in the peripheral blood, and significant coagulation abnormalities, among others [2]. The purpose of this review is to focus on the role of hematology laboratories in two common emergencies—thrombotic microangiopathic anemia (TMA) and acute promyelocytic leukemia (APL). These emergencies aptly highlight the complexity of laboratory workflow, including automation, morphologic analysis, and specialized testing, while underscoring the importance of clinical correlation and detailing pertinent diagnostic advancements.

## 2 Thrombotic Microangiopathic Anemias

TMAs encompass a group of life‐threatening conditions where early recognition plays a crucial role in guiding specific treatments that significantly improve prognosis and survival. These conditions primarily include thrombotic thrombocytopenic purpura (TTP) and hemolytic uremic syndrome (HUS). In addition, disseminated intravascular coagulation (DIC) has some similarities of schistocyte formation pathogenesis. TTP, HUS, and DIC are considered true medical emergencies as they have a common feature—microvascular thrombosis—which leads to thrombocytopenia, schistocytosis, and eventual end‐organ damage [3].

The morphologic hallmark of TMA is the presence of schistocytes. Schistocytes—a relatively frequent finding seen in a hematology laboratory—can be associated with many pathologic conditions including emergencies. The schistocyte identification by a laboratory may be the earliest warning of TMA [4]. Ensuring that laboratory professionals apply clear morphologic criteria for schistocyte identification, understand the clinical implications of this finding, and establish a framework for accurate reporting is critical for the timely workup of these emergencies.

Schistocytes result from the fragmentation of red blood cells (RBC) in circulation. A common mechanism of such fragmentation is the passage of RBCs through small blood vessels (e.g., arterioles or capillaries) that have interlacing fibrin strands across their lumen. As the RBCs pass, they are cleaved and deformed, forming irregular RBC fragments. This mechanism is responsible for schistocyte formation in TMAs, where the formation of platelet‐rich microthrombi is one of the key features.

RBC fragmentation can also occur due to increased turbulence of blood flow. This mechanism occurs in vascular malformations and abnormalities of large vessels or valves, including artificial cardiac valves or cardiac‐assisted devices. Additionally, increased RBC disruption may result from abnormalities in the RBC membrane. In burn patients, exposure to extremely high temperatures can denature spectrin, a key RBC protein that stabilizes cell shape, leading to significant fragmentation. On the other hand, germline mutations in genes coding for membrane proteins can result in a hereditary RBC membrane disorder known as hereditary pyropoikilocytosis.

Another mechanism of RBC fragmentation is associated with intracellular hemoglobin (Hb) denaturation, occurring due to an intrinsically unstable Hb variants or faulty enzymatic machinery (e.g., glucose‐6‐phosphate dehydrogenase deficiency). In these conditions, Hb precipitates and adheres to the cell membrane. In the spleen, the precipitated Hb is removed, resulting in a shape defect. This process classically produces “bite cells” and “double bite/apple core cells,” and other ambiguous RBC fragments mimicking the appearance of “true” schistocytes.

Schistocytes are morphologically defined as smaller RBC fragments (when compared to intact erythrocytes). They typically exhibit a homogeneous staining pattern and lack the central pallor seen in normal RBCs. Schistocytes generally present with sharp angles and straight borders, although small crescents, helmet cells, and keratocytes—each showing a loss of central pallor—may fall into the category of schistocytes in an appropriate clinical context. Microspherocytes, small round RBCs without central pallor, are included in a schistocyte count only when other forms of schistocytes are present as well [4].

To achieve a reliable schistocyte count, it is recommended that the blood smears are prepared from specimens stored for no longer than 3 h at ambient temperature or 8 h at 4°C. The percentage of schistocytes should be expressed as a fraction of the total RBCs, based on the review of at least 1000 RBCs. Recommendations suggest schistocyte evaluation at a magnification of at least 400× [4].

Routinely used automated hematology analyzers can assist in the identification of schistocytes, or, more precisely “fragmented RBCs” (FRBCs). This identification is based on the multiparametric evaluation of RBCs. For example, Sysmex cell counters can identify FRBCs as a subset of RBCs with a decreased high‐angle forward scatter, which corresponds to their smaller size. Platelets, which often have overlapping forward scatter characteristics with FRBCs, can be differentiated by a higher fluorescent intensity, reflecting the differences in cellular content. Furthermore, Sysmex analyzers use fluorochrome staining of ribosomal RNA, and in turn combine RNA content (lower in FRBCs compared to platelets) and forward scatter data to isolate the FRBC fraction.

Although automated analyzers can provide reliable recognition of FRBCs, they fail to produce quantitative data that matches manual assessment. For example, when compared to the “gold standard” manual schistocyte enumeration in patients with suspected TMA or thalassemia, the Sysmex XN‐3000 analyzer's ROC curve showed an area under the curve of 0.803, indicating a reasonable concordance in schistocyte identification. However, there is considerable disagreement between automated and manual enumeration [5]. A comparison of the ADVIA 120 analyzer to manual enumeration showed less reliable FRBC detection (correlation coefficient of 0.7274) and the tendency of the instrument to overestimate the FRBC fraction [6].

When automated analyzers are used for FRBC assessment, all flagged samples must undergo manual review to confirm and properly enumerate schistocytes. Generally, the absence of FRBCs by hematology analyzers can help exclude schistocytosis, except in samples with high mean corpuscular volume (MCV). Therefore, peripheral smears with high MCV and a clinical suspicion for TMA should be manually reviewed, irrespective of FRBC flagging by an analyzer.

The implementation of automated image analysis may provide workflow benefits. For instance, CellaVision Advanced RBC software has demonstrated reasonable sensitivity and reproducibility in enumerating schistocytes in peripheral blood. However, its lower specificity necessitates manual review of preclassified RBC subsets [7]. The further implementation of artificial intelligence (AI) will likely provide significant advantages. One such AI‐based decision support system, developed by Scopio Laboratories (Tel‐Aviv, Israel), can examine over 10 000 cells in peripheral blood smears while assessing 22 parameters for RBCs. This approach seems quite promising and may limit laborious manual reviews.

### 2.1 Thrombotic Thrombocytopenic Purpura

TTP is caused by marked deficiency in the enzyme ADAMTS13, which can be hereditary or, more commonly, acquired. ADAMTS13 is the principal agent limiting formation of large multimers of von Willebrand factor (vWF)—a potent facilitator of platelet adhesion, activation, and aggregation. Inactivation of ADAMTS13 allows polymerization of vWF and widespread microthrombi formation leading to consumptive thrombocytopenia and multiorgan damage, which are characteristic features of the disease [8].

In most cases, deficiency of ADAMTS13 is caused by acquired autoantibodies against ADAMTS13, demonstrated by positive anti‐ADAMTS13 IgG during the acute phase of TTP [8]. These autoantibodies generally inhibit the proteolytic activity of ADAMTS13. In 10%–25% of cases, inhibitor antibodies are not identified, possibly due to an increased clearance of ADAMTS13. Rarely (approximately 5%), TTP is congenital and results from recessive mutations in the *ADAMTS13* gene.

Patients with TTP commonly present with fever, renal, and neurologic dysfunction. Laboratory workup shows hemolytic anemia, increased schistocytes, and thrombocytopenia. Distinction of TTP from other TMAs may be challenging at this stage. Nonetheless, increased schistocytes in this clinical setting should raise a suspicion of TTP and drive subsequent laboratory workup.

A specific laboratory testing must include ADAMTS13 activity and inhibitors/anti‐ADAMTS13 IgG measurements [9]. Severe ADAMTS13 deficiency (< 5%–10% activity) is virtually diagnostic of TTP. However, some TTP patients may have only slightly decreased ADAMTS13 activity, in which case it is crucial to exclude HUS and other secondary causes of TMA. Several score models are available to predict the likelihood of TTP with severe ADAMTS13 deficiency. Examples include the PLASMIC score and French score, which scrutinize factors such as platelet count, serum creatinine level, laboratory indices of hemolysis, and clinical history [10, 11].

The therapeutic strategy for TTP aims to restore ADAMTS13 activity and remove large vWF multimers. This is achieved by therapeutic plasma exchange in conjunction with corticosteroids, possibly anti‐CD20 (rituximab) and anti‐vWF agents (caplacizumab) [12, 13]. Early diagnosis and prompt treatment have significantly improved the survival rate of TTP patients, increasing it from 10%–20% to much higher (80%) rates with appropriate management.

### 2.2 Hemolytic Uremic Syndrome

HUS is a rare disease which includes two pathogenetically distinct entities: Shiga toxin‐associated HUS (STEC‐HUS) and atypical HUS (aHUS). The former and most common form is often preceded by prodromal diarrheal illness. Shiga/shiga‐like toxins, produced by several enterobacteria including *Escherichia coli* and *Shigella dysenteriae* , permeate the intestinal wall and enter the circulation. These toxins cause endothelial injury in renal glomeruli and cerebral vessels due to their expression of Gb3 receptors, resulting in thrombocytopenia, TMA, and signs of acute kidney injury and neurologic symptoms in severe cases. STEC‐HUS diagnosis is based on the clinical presentation, as well as the identification of Shiga toxin‐producing microorganisms in stool cultures and by molecular testing. Routine laboratory tests may demonstrate general features of TMA (elevated LDH, low haptoglobin), including increased schistocytes. Occasionally, elevated D‐dimer and soluble fibrin degradation products are present, but usually not as high as in DIC; intact ADAMTS13 activity distinguishes STEC‐HUS from TTP. The treatment for STEC‐HUS is not standardized and is usually supportive, focusing on managing symptoms and organ dysfunction.

aHUS shares many clinical and laboratory features with STEC‐HUS, but results from abnormal activation of the alternative complement pathway. aHUS can be triggered by mutations in the genes encoding factors of the complement system (inherited or sporadic) or the development of autoantibodies causing inactivation of these factors. That results in a deregulated and overactivated complement cascade, ultimately introducing injury to endothelial cells and complement‐driven platelet activation. In aHUS, ADAMTS13 levels are typically normal or only mildly decreased. Currently, available clinical tests to assess the complement system are often noncontributory to the diagnosis of aHUS. For example, serologic tests for C3, C5, C5b‐9, and measurements of factors H and I have shown unsatisfactory diagnostic sensitivity and specificity in the context of aHUS [14]. Therefore, aHUS is a diagnosis of exclusion—after secondary TMA, TTP, and STEC‐HUS have been ruled out [15]. Treatment of aHUS may include plasma exchange and an anti‐C5 agent (eculuzimab). The use of the latter requires exclusion of possible TTP (ADAMTS13 testing) and prophylaxis against encapsulated microorganisms (*Neisseria* and *Haemophilus*).

### 2.3 Disseminated Intravascular Coagulation

DIC is a pathogenetically complex consequence of many acute and chronic conditions. It is characterized by excessive activation of the coagulation cascade, leading to thrombin formation, increased and imbalanced consumption of coagulation factors and platelets, and a compensatory increase in fibrinolysis. Fibrin‐rich thrombi in microcirculation are responsible for the formation of schistocytes. Diagnosing DIC can be challenging due to its complexity, which is reflected in the use of scoring systems aimed at improving diagnostic accuracy.

Key to the diagnosis of DIC is the demonstration of increased fibrinolysis in the context of coagulopathy and thrombocytopenia, although the latter is not always present. Laboratory tests such as D‐dimer, fibrinogen degradation products, and soluble fibrin monomers are all useful indicators of fibrinolysis in DIC evaluation. The activation of coagulation leading to consumption of coagulation factors can be demonstrated by prolonged clotting times, decreased levels of fibrinogen and other factors, including factor VIII.

The diagnostic role of schistocytes in DIC remains controversial. Schistocytes are frequently seen in blood samples from patients with DIC, but their numbers are variable and often fall below the recommended 1% threshold for clinical significance [16]. Therefore, while the presence of increased schistocytes in an appropriate clinical setting may raise a consideration for DIC, they are neither a sensitive nor specific indicator of DIC.

### 2.4 Other Conditions With Increased RBC Fragments

Aside from the discussed entity, the identification of schistocytes or their mimickers raises a broad differential diagnosis. A careful review of a blood smear can often allow a morphologist (laboratory scientist, hematopathologist, or hematologist) to identify the underlying pathogenic mechanism of RBC fragmentation. Potential morphologic overlap between keratocytes and “bite cells” may create confusion between schistocytosis and Heinz body hemolytic anemia or nonspecific anisopoikilocytosis. A blood smear from patients with Heinz body anemia does not have triangular fragments.

Often, schistocytes represent a minor fraction of poikilocytes. To eschew excessive counting and reporting of schistocytes in non‐clinically relevant conditions, the schistocyte percentage should only be reported when they constitute a prominent abnormality in the smear. The clinically significant cutoff for schistocytes is 1% in adults and full‐term neonates, and 5% in preterm neonates [4]. Notably, the absence of schistocytes should not be employed as an exclusion criterion of relevant clinically suspected conditions.

**FIGURE 1:**
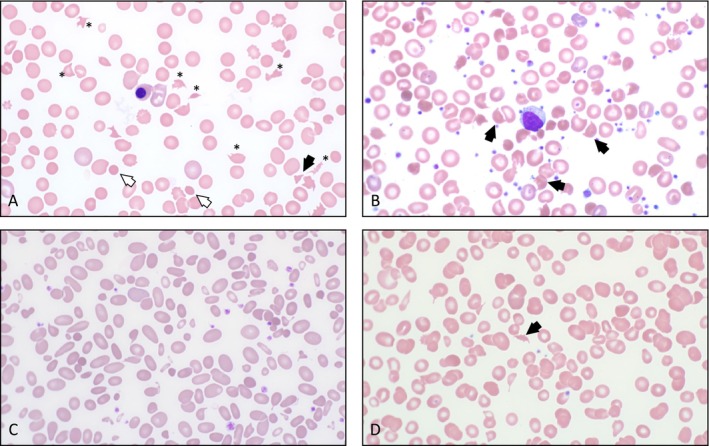
Schistocytes and their mimickers. (A) Numerous schistocytes (asterisk) including “triangulocytes” (black arrow) and microspherocytes (white arrow) along with increased polychromasia, nucleated erythrocyte, and thrombocytopenia in a patient with thrombotic thrombocytopenic purpura. (B) Blood smear from a patient with unstable hemoglobin (Heinz body anemia) shows numerous poikilocytes with many “bite/helmet” cells (black arrows). The platelet number appears adequate. (C) Membrane protein defects may produce numerous poikilocytes that can be mistaken for schistocytes. Although erythrocyte fragments are present in this smear from a patient with hereditary pyropoikilocytosis, they comprise a minor subset of other poikilocytes (ovalocytes and elliptocytes in this case). (D) Poikilocytosis with schistocytes can be seen in many conditions. This patient with vitamin B_12_ deficiency has occasional poikilocytes (black arrow) and borderline thrombocytopenia, which may raise a suspicion for thrombotic microangiopathic anemia.

RBC fragmentation can be seen in many other settings such as hereditary pyropoikilocytosis, thalassemia, vitamin B_12_ deficiency, and thermal injuries, etc. (Figure 1). However, if TMA is suspected, it is crucial to urgently notify the clinical team for further evaluation and management (Table 1).

**TABLE 1:** Pathologic conditions associated with increased schistocytes.

Mechanism of red blood cell fragmentation	Pathologic conditions		Key laboratory tests
	Group	Entity	
Passage through fibrin strands/thrombi in microcirculation	Thrombotic microangiopathic anemia	Thrombotic thrombocytopenic purpura (TTP)	ADAMTS13 activity, ADAMTS13 inhibitor, including anti‐ADAMTS13 IgG
		Shiga toxin‐associated hemolytic uremic syndrome (STEC‐HUS)	Stool culture, Shiga toxin stool PCR
		Atypical hemolytic uremic syndrome (aHUS)	Complement factors, if appropriate
		HELLP‐syndrome	No specific testing
		Hematopoietic stem cell transplant‐associated	
		Malignancy‐associated	
	Disseminated intravascular coagulation (DIC)		D‐dimer, soluble fibrin monomers, fibrin degradation products
Increased turbulence of blood flow	Artificial cardiac valves, cardiac devices, cardiac valve stenosis, malignant hypertension		No specific testing
Removal of denatured intracellular hemoglobin (Heinz body)	Acquired	Some toxins and drugs	No specific testing
	Inherited	Unstable hemoglobin	Hemoglobin fractionation and molecular confirmation
		Glucose‐6‐phosphade dehydrogenase deficiency	Enzyme activity and molecular confirmation
Defects of red blood cell membrane	Inherited	Hereditary pyropoikilocytosis, etc.	Osmotic fragility, ektacytometry, flow cytometry for Band3, molecular confirmation
Other	Inherited	Sickle cell anemia	Hemoglobin fractionation and molecular confirmation
		Congenital dyserythropoietic anemia (CDA)	Molecular
	Acquired	Vitamin B_12_ deficiency, iron deficiency	Depends on the suspected entity
		Infections, for example, COVID‐19, sepsis	
		Splenic angiosarcoma	

## 3 Acute Promyelocytic Leukemia

APL is a relatively rare disease, comprising only 5%–13% of AMLs in adults [17]. Although acute leukemias generally require immediate medical attention, APL constitutes a true hematologic emergency. High pre‐treatment mortality along with excellent post‐treatment cure rates underscore the urgency and gravity to rapidly diagnose APL.

The unique therapeutic approach to APL is determined by the characteristic molecular mechanism of the disease. The hallmark of APL is rearrangement of the *RARA* (retinoic acid receptor‐α) gene on chromosome 17, which encodes for the nuclear hormone receptor transcription factor. A translocation between chromosomes 15 and 17—t(15;17)(q22;q21)—identified in 95% of APL cases, results in a fusion of the *RARA* gene with the *PML* (promyelocytic leukemia) gene. The resultant chimeric protein binds to histone deacetylase with high affinity and blocks the differentiation of myeloid/granulocytic precursors at the promyelocytic stage [18]. All‐trans retinoic acid (ATRA) at a therapeutic concentration alleviates this inhibition, allowing the neoplastic immature cells to mature into neutrophils. The therapeutic addition of arsenic trioxide (ATO) induces apoptosis of the neoplastic cells. The combination of ATRA and ATO has transformed APL from a deadly to curable disease in most patients, with long‐term survival approaching 90% [18, 19].

Clinically, APL often initially manifests as acute coagulopathy. This pathogenetic phenomenon occurs when tissue factors, expressed by neoplastic promyelocytes, interact with factor VII and subsequently activate factors X and IX, resulting in hypercoagulation followed by activation of fibrinolysis and a hypocoagulable state. Nearly all patients diagnosed with APL present with coagulopathy, which can lead to numerous complications, often life‐threatening DIC [20].

APL is primarily a disease of young patients, although cases may also present in elderly populations [17]. Patients usually present with fatigue, weakness, as well as pancytopenia caused by marrow replacement by neoplastic cells. This marrow involvement, combined with coagulopathy, may lead to thrombocytopenia, which manifests as epistaxis, retinal hemorrhages, and menorrhagia. Bleeding (mostly due to hyperfibrinolysis) poses a significant risk of mortality. Additionally, some patients present with thrombotic complications, such as pulmonary embolism or cerebrovascular complications. In a cohort of 105 patients with APL in Sweden, death within the first 30 days of presentation occurred in 29% of individuals, highlighting the time‐sensitive nature of APL diagnosis. Of those, 41% succumbed due to hemorrhage, with an alarming 35% of patients succumbing to early death not receiving ATRA [21].

The definitive diagnosis of APL requires a demonstration of increased atypical promyelocytes in the peripheral blood and/or bone marrow (≥ 10% by ICC; no set cutoff by WHO 5th) along with detection of a translocation affecting the *RARA* gene such as t(15;17) or a variant translocation, and no history of cytotoxic therapy (specified in WHO, 5th edition) [22]. Rarely, APL can arise in patients with prior cytotoxic therapy. By WHO, 5th edition, such cases would be designated as “myeloid neoplasms post cytotoxic therapy (AML‐pCT)”. APL‐pCT is associated with prior administration of DNA topoisomerase II inhibitors and is treated similarly to conventional APL [23]. Variant translocations are rare, but several partner genes have been described: *ZBTB16* at 11q23, *NUMA1* at 11q13, *NPM1* at 5q35, *STAT5B* at 17q21, etc. Such cases are diagnosed as “APL with a variant *RARA* translocation” in contrast to “APL with *PML::RARA* fusion”.

The successful treatment and eventual outcome of APL depend on a timely diagnosis. Abnormal promyelocytes—the key morphologic feature of APL—place hematology laboratories at the frontline of the diagnostic process. Since the broad clinical presentation of APL makes its recognition clinically challenging, hematology laboratories often raise the initial suspicion for APL.

Results of an automated CBC in APL patients vary, but pancytopenia is frequent. While the white blood cell (WBC) count may be increased, it is often decreased with absolute neutropenia. Modern hematology analyzers have variable capabilities to detect promyelocytes. In some systems, a relative increase in promyelocytes triggers an “immature granulocytes” or “left shift” flag. In other analyzers, particularly those evaluating myeloperoxidase (MPO), promyelocytes can be detected by an abnormal pattern of MPO fluorescence. Other “flags” may include thrombocytopenia, leukocytosis, increased fragmented RBCs, etc. It is imperative that laboratories establish a process where flags commonly associated with APL prompt the preparation of blood smears, followed by a detailed morphological review of the slides.

The neoplastic cells in APL—granulocytic precursors with arrested maturation at the promyelocyte level—are typically detectable in the peripheral blood of nearly all patients at presentation. These cells may resemble normal promyelocytes but often exhibit features reflecting their abnormal nature. The distinctive morphologic feature of promyelocytes is primary granules within their cytoplasm. In well‐stained smears by Giemsa‐based methods, primary granules are azurophilic (dark purple) and usually more conspicuous than the secondary granules, which are finer and stain lilac to pale‐orange or pink. Promyelocytes constitute a minor subset of myeloid cells in aspirate smears from healthy individuals, typically comprising 1%–3% of the total. They are characterized by their relatively large size (20–25 μm), higher nuclear‐to‐cytoplasmic ratio (though lower than that of blasts), and round to oval nuclei with immature chromatin and often nucleoli. Promyelocytes have a blue cytoplasm that contains primary granules of varying size and number depending on their stage of maturation. Occasionally, a paranuclear clearing, a so‐called perinuclear hof corresponding to the Golgi apparatus, can be appreciated. Promyelocytes are not typically observed in the peripheral smears of healthy individuals. Their presence is often associated with a granulocytic left shift, which may be reactive or neoplastic (e.g., chronic myeloid leukemia). However, when promyelocytes are identified outside the context of a left shift, they should prompt suspicion for APL.

**FIGURE 2:**
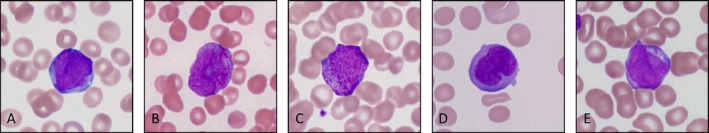
Morphologic spectrum of neoplastic cells in acute promyelocytic leukemia and its mimickers. (A) “Classic” *PML::RARA*‐positive acute promyelocytic leukemia. The atypical promyelocyte has morphologic features of a blast. Although the cytoplasm lacks prominent granules, a single slender Auer rod is present. (B) Another case of *PML::RARA*‐positive acute promyelocytic leukemia. The atypical promyelocytes contain more abundant cytoplasm and primary granules with numerous Auer rods. (C) Acute promyelocytic leukemia with an alternative *RARA* translocation: t(11;17) *ZBTB16::RARA*. The promyelocyte shows heavily granulated cytoplasm devoid of Auer rods. (D) Microgranular variant of *PML::RARA*‐positive acute promyelocytic leukemia. The neoplastic promyelocyte demonstrates a “sliding disks” nucleus and absence of cytoplasmic granules. This appearance may raise a differential diagnosis of monocytic differentiation. (E) A myeloblast in a patient with acute myeloid leukemia with mutated *NPM1*. The morphology of the neoplastic cells, including an Auer rod, is very similar to that of acute promyelocytic leukemia.

The two most common morphologic variants of promyelocytes in APL include hypergranular (classic) and hypogranular (microgranular), corresponding to two APL variants. In a hypergranular variant, the promyelocytes contain easily identifiable primary granules in the cytoplasm, often tightly packed and obscuring the nucleus. The nuclei are round, oval, or irregular with immature chromatin. Along with promyelocytes, blasts (immature cells devoid of granulation) are also frequently encountered. A common finding in hypergranular APL is Auer rods (Figure 2A)—elongated generally rod‐shaped cytoplasmic crystalline structures resulting from fusion of primary granules. The shape of Auer rods can vary from slender with tapered ends to short and diamond‐shaped. Auer rods may appear as solitary structures or in multiples, frequently clustering into bundles (Figure 2B). Auer rods are definitive indicators of the abnormal/neoplastic nature of a cell and signify myeloid differentiation. The presence of blasts and/or promyelocytes with Auer rods should raise suspicion for APL. However, Auer rods are not specific to APL, as they can also be observed in other myeloid malignancies, including acute myeloid leukemia subtypes and myelodysplastic syndromes/neoplasms (Figure 2E). A microgranular variant of APL denotes paucity of morphologically identifiable primary granules in neoplastic promyelocytes. These cells are characterized by a relatively low nuclear‐to‐cytoplasmic ratio due to abundant cytoplasm. Their nuclei are often bilobed or folded, featuring a prominent slit‐like indentation. Due to this distinctive morphology, the nuclei are commonly described as “sliding disks” or “butterfly wings” (Figure 2D). The “hypogranular promyelocytes” may resemble monoblasts, which poses a diagnostic hurdle. Although not readily visible under a light microscope, hypogranular promyelocytes retain primary granules detectable at the ultrastructural level. These granules can be confirmed by their strong positive staining for MPO. Alternatively, butyrate esterase (nonspecific esterase), a monocytic marker, is negative or weakly positive. In contrast, monoblasts exhibit prominent butyrate esterase staining. Upon diligent examination of peripheral blood smears, well‐granulated promyelocytes and/or Auer rods can be discerned even in hypogranular APL. Rare cases of APL have been reported in patients with hereditary MPO deficiency. In these instances, the neoplastic cells exhibit weak MPO expression, which can complicate the diagnosis. However, recognizing diminished MPO staining in the background mature neutrophils can help identify this rare etiology [24].

Along with hyper‐ and microgranular variants, a bevy of less common morphologic abnormalities in promyelocytes have been described; “hyperbasophilic” promyelocytes or cells with eosinophilic granules among these variations. An association between morphology and molecular/genetic profile has also been reported; in the most common variant *RARA* translocation, t(11;17) *ZBTB16::RARA*, circulating promyelocytes often present as heavily granulated, rarely display Auer rods, and demonstrate an increased prominence of irregular nuclear contours (Figure 2C).

In any given case, neoplastic promyelocytes in circulation demonstrate a broad morphologic spectrum. For diagnostic purposes, any cells resembling promyelocyte must be counted as a “blast equivalent” and be added to the blast count. The diagnosis of APL can be made in the presence of characteristic cytogenetic or molecular findings, even if the blast or blast‐equivalent count is less than 20% [22]. Cases with the *PML::RARA* fusion and fewer than 10% blasts or promyelocytes are exceedingly rare and are likely indicative of early‐stage APL [25].

Any reasonable suspicion for APL raised by the clinical laboratory must be urgently communicated to the health care provider. Specific therapy must be initiated without delay, even before cytogenetic confirmation is readily available. Additionally, suspicion of a myeloid neoplasm triggers further testing, including flow cytometry (FC), cytogenetic studies (such as karyotyping and fluorescence in situ hybridization [FISH]), and molecular analysis. In many cases, a bone marrow biopsy is also performed. Given the elevated risk of DIC, all patients with suspected APL must be promptly tested for at least routine coagulation parameters, including prothrombin time (PT), activated partial thromboplastic time (aPTT), d‐dimer, and fibrinogen.

Recognizing the essential role of morphology in diagnosing APL, efforts have been underway to develop deep‐learning methods to assist hematologists and hematopathologists. Shenderov et al. demonstrate a successful prediction of APL by their AI algorithm based on images generated by CellaVision with a potential use of “a cloud application on a smartphone mounted to a microscope” [26].

FC is a rapid and valuable tool in the workup of AML [27]. Even when reviewed independently of morphology (e.g., in a reference laboratory setting), it can help identify cases with a high probability of APL. Abnormal promyelocytes are characterized by moderate to dim CD45 expression and variable side scatter characteristics, which correspond roughly to the granularity of the cytoplasm (hypergranular promyelocytes exhibit higher side scatter than microgranular ones). The “classic” APL immunophenotype is positive for CD117, bright CD33, and MPO while negative for CD34 and HLA‐DR [28]. CD64 is also commonly expressed [28, 29]. Microgranular APL may show CD2 expression and a higher frequency of CD34 and HLA‐DR positivity, although CD34 and/or HLA‐DR positivity are rare. An APL‐like immunophenotype is seen in a significant number of patients with acute myeloid leukemia (AML) with *NPM1* mutation (approximately 30%) and AML with monocytic differentiation [30]. Uniform expression of CD13 and CD64 positivity can help distinguish APL from AML with *NPM1* mutations. Another APL look‐alike, AML with *KMT2A* rearrangement, can be differentiated from APL by partial or variable MPO positivity compared to strong positivity in APL [28, 31]. FC analysis of an APL with variant *RARA* fusion partners is characterized by more frequent CD56 expression, but otherwise closely resembles APL with *PML::RARA* [32].

Cytogenetic studies provide a definitive diagnosis of APL. Methodologies for detection of the most common t(15;17) translocation may include targeted (FISH) and general (karyotype) cytogenetic studies. Both modalities offer distinct advantages and disadvantages, and therefore, should be performed in tandem. The primary advantage of FISH is its rapid performance, which significantly shortens turnaround time compared to conventional cytogenetic studies. Additionally, because FISH can be performed on interphase cells, it does not require the time‐consuming process of culturing or mitogenic stimulation. Clinically offered t(15;17) FISH should be performed and resulted within 24 h. Some disadvantages of FISH are inherent to the test design and methodology. Since only a few chromosomal segments, such as *PML* and *RARA*, are targeted, FISH can only assess these specific regions, which limits its ability to identify cases with variant *RARA* rearrangements. Additionally, FISH does not detect other chromosomal abnormalities that may be present. The sensitivity of FISH also depends on the types and sizes of the probes selected, which can impact its overall accuracy and detection capabilities [33]. Some institutions, in addition to a dual‐color fusion probe (*PML* and *RARA*) also perform dual‐color break‐apart probe targeting *RARA*, which increases the diagnostic sensitivity. Conventional karyotyping circumvents the mentioned drawbacks of FISH. The main shortcoming is the turnaround time, which exceeds 3 days and usually is about 7 days. Another potential drawback of conventional cytogenetics is its lower diagnostic sensitivity, as typically only 20 cells are karyotyped. Poor banding quality or complex chromosomal aberrations can significantly limit the effectiveness of karyotyping. Additionally, cryptic *PML::RARA* rearrangements pose a challenge for both FISH and conventional cytogenetics, as these cases may show no identifiable cytogenetic abnormalities by either method, yet the *PML::RARA* fusion can still be demonstrated through molecular analysis [34, 35, 36]. Additional cytogenetic aberrations occur in approximately 25%–40% of patients, with trisomy 8 being the most common one.

Quantitative reverse transcription‐polymerase chain reaction (qRT‐PCR) is an essential part of the standard diagnostic workup for APL. In addition to providing rapid (< 24 h) diagnostic information about the presence of PML::RARA transcripts, it is suitable for minimal residual disease (MRD) evaluation, with a limit of detection of 1 in 10 000 cells. However, qRT‐PCR is limited by its inability to detect alternative *RARA* gene partners. The formation of the three PML::RARA transcript subtypes depends on the location of breakpoints within the *PML* gene (intron 6, exon 6, and intron 3) and within the *RARA* gene (intron 2). These subtypes—long (L or bcr1), variant (V or bcr2), and short (S or bcr3)—account for 55%, 5%, and 40% of cases, respectively [37].

The need for rapid molecular testing for *PML::RARA* fusion, especially in low‐ and middle‐income settings where more precise molecular testing poses challenges, has stimulated a search for alternative approaches. For example, Yeung et al. have optimized an assay to detect common gene fusions, including *PML::RARA*, using a rapid sequencing technology based on nanopores [38]. Alternatively, detection of PML::RARA protein by immunocytochemistry can be a rapid and cost‐effective solution [39].

Approximately 70% of patients have at least one mutation in addition to *PML::RARA* [40]. Common mutations include activated signaling pathways, for example, *FLT3‐ITD* (found in 41% of cases) and *FLT3‐TKD*, followed by genes involved in DNA methylation, such as *WT1* and *ASXL1*. Less common mutations include *NRAS*, *KRAS*, *ARID1A/1B*, *ETV6*, *DNMT3A*, *RUNX1*, *CBL1*, *SF3B1*, *EZH2*, *CEBPA*, and *TET2*. Approximately 25% of patients develop resistance to ATRA and/or ATO therapy due to mutations that result in amino acid substitutions in the RARA ligand‐binding domain (LBD) or the PML‐B2 domain of PML‐RARA [40].

## 4 Conclusion

Undoubtedly, hematology laboratories play a pivotal role in recognizing, documenting, and reporting findings associated with numerous hematologic emergencies. Timely communication of these findings to the clinical team—often accompanied by consultations and recommendations for subsequent testing—ensures an accurate diagnosis, leading to appropriate treatment and improved outcomes. As laboratories integrate technological advances—ranging from automated analyzers and image acquisition systems to AI‐based algorithms—technologists and pathologists are increasingly handling more sophisticated information, leading to changes in current diagnostic workflows. This evolution demands strong decision‐making skills from laboratory professionals and underscores the importance of training and expertise in technology, morphology, and clinical judgment.

## Ethics Statement

The authors have nothing to report.

## Consent

The authors have nothing to report.

## Data Availability

Data sharing is not applicable to this article as no new data were created or analyzed in this study.
